# Health-system drivers of outpatient antibiotic use: evidence from prescribing, centralized supply, and catchment populations in public tertiary hospitals in Bihar, India

**DOI:** 10.3389/frhs.2026.1800794

**Published:** 2026-06-18

**Authors:** Vinay Modgil, Sundeep Sahay, Ravikant Singh, Arunima Mukherjee, Rashmi Surial, Bibekananda Bhoi

**Affiliations:** 1 Society for Health Information Systems Programmes (HISP India), New Delhi, India; 2 Department of Informatics, University of Oslo, Oslo, Norway; 3 Homi Bhabha Cancer Hospital and Research Centre, Muzaffarpur, India; 4 Institute of Health and Society (HELSAM), University of Oslo, Oslo, Norway

**Keywords:** antibiotic prescribing, antibiotic supply chain, antimicrobial stewardship, tertiary care hospitals, WHO AWaRe classification

## Abstract

**Background:**

In low- and middle-income countries (LMICs), antibiotic use is shaped not only by clinical decision-making but also by health-system factors such as centralized drug procurement, supply availability, and population catchment pressures. However, empirical evidence linking outpatient prescribing patterns with state-level antibiotic supply and catchment characteristics remains limited. Bihar, one of India's most populous and resource-constrained states, represents a critical yet under-studied setting for understanding these interactions.

**Methods:**

We conducted a multicentre observational study across three public tertiary care hospitals in Bihar, SKMCH Muzaffarpur, DMCH Darbhanga, and JLNMCH Bhagalpur, using outpatient prescription data collected between 2023 and 2025 (*n* = 8,822). These data were triangulated with state-level antibiotic supply records from the Bihar Medical Services and Infrastructure Corporation Limited (BMSICL) for 2022–2025 and secondary data describing hospital catchment populations. Prescribing indicators, WHO AWaRe classification, essential drug list (EDL) status, and supply–prescription concordance were analysed to assess alignment with antimicrobial stewardship goals.

**Results:**

Across all sites, outpatient antibiotic use was characterized by high polypharmacy (4–4.7 drugs per prescription), extensive empirical prescribing, and minimal use of culture-based diagnostics (≤2.6%). Broad-spectrum antibiotics dominated prescribing, with Amoxicillin–Clavulanic acid (42.8%), Azithromycin (13.1%), and Cefixime (6.9%) being most frequently used. While Access-category antibiotic use exceeded 50% at two sites, Watch-category use was disproportionately high at JLNMCH (58%), a hospital serving an extensive rural catchment with long patient travel times. Supply–prescription analysis revealed strong alignment for commonly used antibiotics, indicating stable state-level procurement patterns; however, the continued use of Watch-category and non-EDL antibiotics, including Moxifloxacin, raised stewardship concerns. Notably, irrational fixed-dose combinations were prescribed despite not being supplied, indicating parallel private-sector access. Generic prescribing exceeded 97% across all sites, reflecting a positive institutional norm.

**Conclusions:**

Antibiotic prescribing in Bihar's public tertiary hospitals is influenced by the interactions of centralized supply stability, diagnostic constraints, and catchment-level pressures rather than prescriber behaviour alone. Persistent availability of broad-spectrum antibiotics through state procurement may be associated with entrenched prescribing patterns, particularly in high-volume, rural-serving facilities. Effective antimicrobial stewardship in such settings requires context-sensitive strategies that integrate supply-chain reform, strengthened diagnostic pathways, and locally tailored prescriber support, moving beyond uniform, guideline-only approaches.

## 1 Introduction

There is limited understanding of antibiotic prescription patterns in low- and middle-income countries (LMICs), where antibiotics are accessed through multiple channels: formal outpatient and inpatient care, self-medication, over-the-counter (OTC) sales, and informal providers. Most of these transactions remain undocumented, leaving very little systematic information to guide policy. Previous studies have noted that while data from high-income countries are relatively robust, evidence from LMICs remains fragmented, often limited to single-institution studies or private-sector sales audits, with few systematic analyses of public sector use (1, 2). This constitutes a major limitation in the global fight against antimicrobial resistance (AMR), particularly in regions where antibiotic misuse is widespread, and stewardship mechanisms are weak.

**Figure 1:**
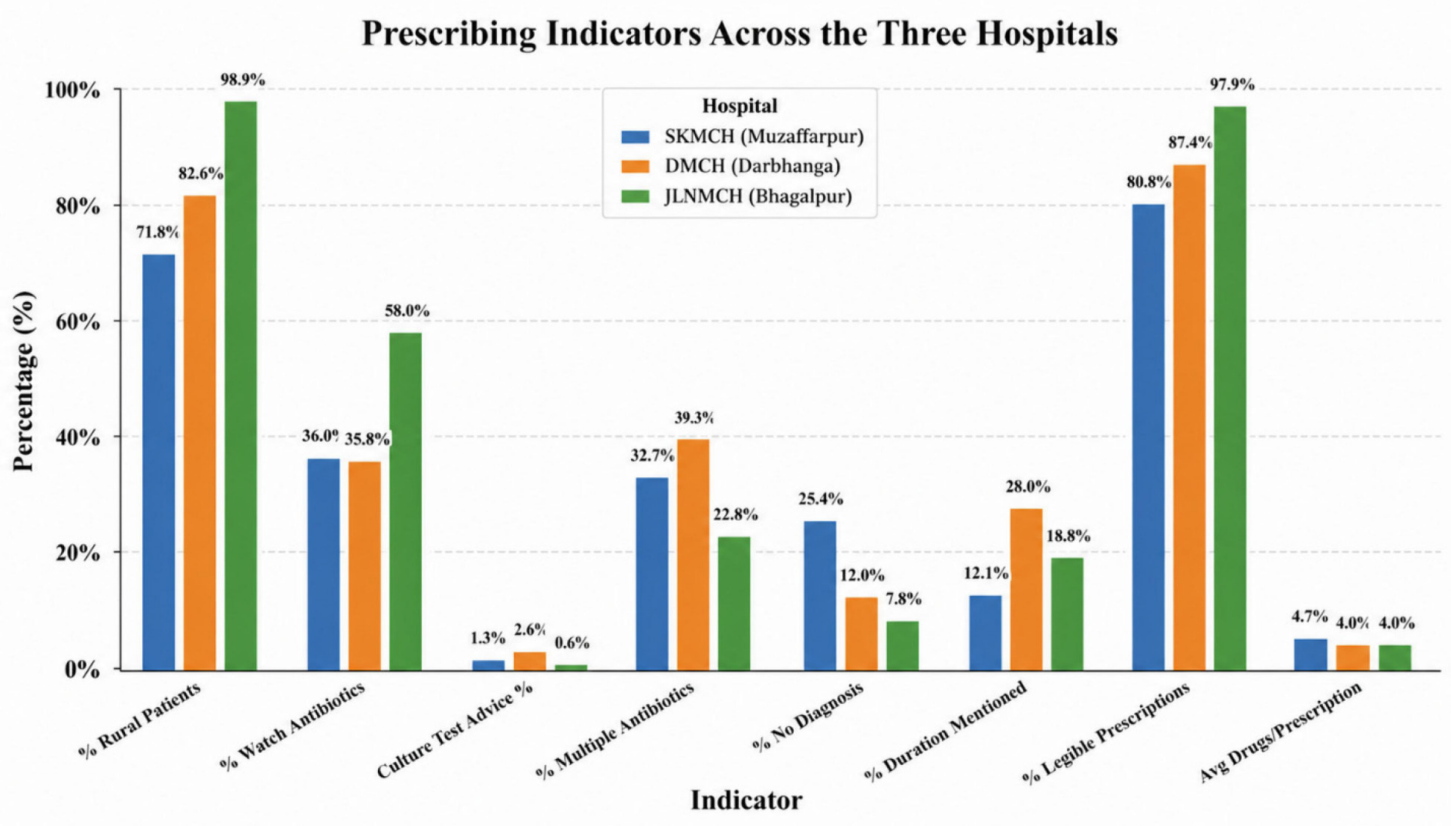
Summary of prescription indicators and percentages.

AMR is among the most pressing public health challenges of the 21st century. The World Health Organization (WHO) has warned that without urgent action, infections once easily treatable with antibiotics could become life-threatening (3). A landmark study estimated 1.27 million deaths attributable to AMR in 2019 and projected up to 10 million annually by 2050 if current trends continue. In India, where public and private healthcare systems coexist with widespread OTC availability of antibiotics, prescription practices are highly variable and often poorly documented, reflecting the broader challenges of LMIC settings (31). Bihar, India's third most populous state with a largely rural and economically disadvantaged population, represents a critical yet under-researched context within this national landscape. Therefore, situating Bihar within the broader AMR discourse is essential, given that AMR is already escalating healthcare costs, extending hospital stays, and worsening health outcomes globally.

Prescription practices are not confined to physician–patient encounters alone but are shaped by parallel influences such as drug supply-driven availability, pharmacy-level substitutions, and informal practices within overcrowded outpatient settings. For instance, studies from India and other LMICs have shown that when essential drugs are out of stock, prescribers may adapt by choosing alternatives available in the hospital pharmacy. At the same time, patients frequently resort to purchasing antibiotics from private chemists, often without prescriptions (4). These fragmented channels create a “pluralistic” prescribing environment where clinical decisions are entangled with systemic gaps, patient expectations, and drug availability. In Bihar, as in many resource-constrained settings, this multiplicity is exacerbated by weak referral systems, lengthy travel times, and patient expectations for comprehensive treatment in a single visit, which further fuels polypharmacy and reliance on broad-spectrum agents. Recognizing these multiple prescribing pathways is essential, as they connect micro-level practices with broader supply and catchment determinants. Together, they provide a more comprehensive lens to explain why irrational prescribing patterns persist despite stewardship frameworks (5, 6).

Global evidence highlights the burden of AMR, but in LMICs, the pathways through which health systems influence antibiotic use, particularly prescribing and drug supply, are less clear. In India, the antibiotics available in public hospitals are largely determined by centralized state procurement systems, but what clinicians can prescribe on the ground is shaped by supplies dispatched by the state to facilities. Misalignments between procurement-supply priorities and stewardship goals, such as the consistent availability of broad-spectrum Watch antibiotics, can perpetuate sub-optimal use (7, 8). Beyond comparing prescribing with hospital-level supplies, it is important to assess whether these choices align with stewardship benchmarks such as the WHO AWaRe classification and India's National Essential Drug List (NEDL). AWaRe categorizes antibiotics into Access, Watch, and Reserve groups, with the WHO recommending that at least 60% of use should come from the Access category. Yet studies from India show persistent over-reliance on Watch antibiotics. Mugada et al. (9) reported high use of Azithromycin and Cefixime in Andhra Pradesh, while Bansal et al. (32) found over 40% of outpatient prescriptions across sites were from the Watch group. Other studies similarly called for improving Access availability and curbing Watch dependence (9, 10, 32). Applying these frameworks to both prescribing and supply patterns requires a stewardship-oriented evaluation of antibiotic use in these settings.

In Bihar, to interpret prescribing and supply patterns meaningfully, it is essential to situate them within the demographic and health system realities. Population characteristics from the Census of India, Annual Health Survey, and NITI Aayog statistics, such as literacy levels, rural–urban mix, disease burden, and health infrastructure, directly shape healthcare-seeking behavior and prescribing pressures. Prior studies have highlighted how patient load, rural catchments, and weak diagnostic capacity influence prescribing practices in India's public hospitals. Despite being India's third most populous state with one of the highest burdens of infectious disease, Bihar has received little systematic research attention on antibiotic use, leaving critical gaps in stewardship planning. This analysis has direct implications for antimicrobial stewardship programs (ASPs), both at the hospital level, where clinicians face overcrowded outpatient departments, limited diagnostics, and the absence of guidelines, and at the state level, where supply and distribution decisions shape access and affordability. In this study, we therefore compare hospital-level prescribing patterns with state-level supply data over three years to identify how the most frequently prescribed antibiotics at the hospital level are aligned with those procured at the state level and supplied to hospitals. We highlight how drug distribution priorities influence clinical decision-making and the pressures faced by tertiary hospitals in terms of catchment population. To our knowledge, this is among the first studies in India to triangulate outpatient prescribing data with centralized drug supply data and catchment demographics, helping to build a health-system perspective on antibiotic use. This helps generate evidence to guide context-sensitive stewardship policies at both the hospital and state levels.

## 2 Materials and methods

### 2.1 Study design and data sources

The study utilized three distinct datasets: (i) outpatient prescription records collected from outpatients during 2023–2025 from three public tertiary hospitals in Bihar (SKMCH Muzaffarpur, DMCH Darbhanga, and JLNMCH Bhagalpur), (ii) state-level antibiotic supply data obtained from the Bihar Medical Services and Infrastructure Corporation Limited (BMSICL) for the financial years 2022–2025, reflecting drugs dispatched to these tertiary hospitals, and (iii) catchment-related demographic and healthcare characteristics of the study sites compiled from secondary government sources.

### 2.2 Study sites

The study was conducted in three sites in Bihar. It was first initiated at Shri Krishna Medical College and Hospital (SKMCH), Muzaffarpur, and subsequently extended to the other two sites, Darbhanga Medical College (DMCH) and Jawaharlal Nehru Medical College (JLNMCH), Bhagalpur. SKMCH is the largest public tertiary care and teaching institute in North Bihar, offering comprehensive care ranging from general outpatient services, emergency services, to super-specialty treatments and cancer care. It is located in Muzaffarpur and is a government-run medical college affiliated with Bihar University. It has an indoor bed capacity of approximately 900 beds. DMCH and JLNMCH institutions also play a vital role in providing healthcare services to their respective catchment populations, in north and eastern Bihar, respectively, as well as in training medical professionals. DMCH is one of the oldest and most prominent medical colleges in Bihar. It functions as a major referral center, offering a range of specialty and super-specialty services. JLNMCH is the primary government medical institution serving Bhagalpur and neighboring districts. All three hospitals serve as important centers for medical education, clinical research, and public health interventions, making them suitable settings for the present study.

### 2.3 Catchment characteristics and healthcare context of study sites

Catchment-related demographic and healthcare indicators for the three study hospitals were compiled from secondary government sources, including the Census of India (11), District Health Portals of Bihar, and the National Health Mission. Extracted variables included the total population served, urban–rural composition, socioeconomic profile, literacy levels, healthcare infrastructure (including PHCs, CHCs, and private clinics), referral pathways, and average patient travel time to these facilities. Table 1 summarizes the catchment population, healthcare infrastructure, and referral patterns across the three hospitals (11–13), each catering to large populations (3.5–5.8 million), predominantly from rural and semi-urban areas, with low to lower-middle socioeconomic backgrounds. Referral patterns differed slightly, with SKMCH and DMCH seeing more walk-ins and private referrals, while JLNMCH mainly receives patients from PHCs and clinics. Patient travel time is often over 2–4 h, particularly from rural areas, reflecting access challenges. Common health issues include infectious diseases, maternal-child health problems, and non-communicable diseases (NCDs), all of which shape outpatient care demand and prescribing pressures at these centers. These secondary data sources were used to provide contextual understanding of the study settings and were not intended to establish direct or contemporaneous explanatory relationships with prescribing patterns.

**Table 1:** Catchment population, healthcare infrastructure, and referral patterns .

Indicator	SKMCH (Muzaffarpur)	DMCH (Darbhanga)	JLNMCH (Bhagalpur)
Population served	∼5.6–5.8 million	∼4.6–4.8 million	∼3.5–3.6 million
Area type	Semi-urban + rural	Semi-urban + rural	Urban + extensive rural
Socioeconomic profile	Low-income, agricultural, migrant workers	Low to lower-middle income, farming + urban workers	Low-income, farming, and daily laborers
Literacy rates	Moderate urban, low rural	Moderate urban, low rural	Slightly lower, esp. rural
Referral pattern	Walk-ins + PHCs + private clinics	PHCs + private referrals + walk-ins	Mostly PHC + clinic referrals
Patient travel time	<1 h (urban), 2–4 h (rural)	1–2 h, some up to 4 h	2–4 h, some >4 h
PHCs & subcenters	102 PHCs, 17 CHCs	90 PHCs, 15 CHCs	65 PHCs, 11 CHCs
Private clinics	Many urban, fewer rural	Urban-focused	Urban, sparse rural
Common health issues	UTIs, respiratory, skin infections, and maternal-child health	Infectious diseases, maternal health, and NCDs	UTIs, skin infections, diarrheal diseases, and maternal complications

Sources: Census of India (11); District Health Portals, Bihar (Government of Bihar); National Health Mission, Bihar.

### 2.4 Prescription data collection

Prescription data were collected over one year at each of the three study sites. At SKMCH, a total of 3,702 prescription slips were analyzed and collected between August 2023 and July 2024; at DMCH, 3,124 prescriptions were reviewed between March 2024 and February 2025; and at JLNMCH, 1,996 prescriptions were studied over nine months from June 2024 to March 2025. Prescriptions were obtained from patients who visited the hospital pharmacy following consultation in the Outpatient Department (OPD). The study purpose was explained to participants in the local language before data collection. Verbal informed consent was obtained from adult patients before collecting prescription details. For patients below 18 years of age, verbal consent was obtained from the accompanying parent or legal guardian. Patients who declined consent were excluded from the study. Prescription information was recorded in a de-identified format, and no patient names or direct personal identifiers were included in the analytical dataset.

A systematic random sampling approach was adopted to enhance representativeness, with approximately 1% of the total daily OPD prescriptions selected for analysis. Sampling was conducted during a fixed high-volume time window (10:00 AM to 1:00 PM), when patient attendance was highest across all sites. Within this period, prescriptions were selected at regular intervals to ensure coverage across different patients and clinical departments. Data collection was carried out on multiple days over the study period at each site, including routine OPD days with varying patient loads. While this approach ensured operational feasibility and captured peak prescribing patterns, it may not fully represent prescribing variations across the entire day or account for temporal (e.g., seasonal) variation. This data were entered into an Excel sheet and subsequently imported into a database for analysis. Within each OPD session, prescriptions were selected using a fixed sampling interval based on patient flow (approximately every 10th–15th prescription), starting from a randomly selected first prescription at the beginning of the sampling period. The sampling frame encompassed all eligible outpatient prescriptions generated during the specified time period. Data collection was conducted on multiple days distributed across the study period at each site, including both high- and moderate-volume OPD days, to improve representativeness of prescribing patterns. No clinical intervention was performed, and no biological samples were collected. All extracted prescription data were de-identified before analysis.

#### 2.4.1 Operational definitions

For consistency and clarity, the following operational definitions were used:

**Empirical prescribing:** Antibiotic prescriptions given based on clinical judgment of the treating doctor in the absence of microbiological confirmation (i.e., no culture or AST results available at the time of prescribing).
**Targeted prescribing:** Antibiotic prescriptions guided by available microbiological evidence, including culture and AST results.
**Prophylactic prescribing:** Antibiotics prescribed to prevent infection rather than to treat an existing infection, based on clinical indication.
**No diagnosis mentioned:** Prescriptions in which no signs, symptoms, or clinical diagnosis were recorded on the prescription slip to justify antibiotic use.
**Prescription legibility:** Prescriptions were classified as legible if all key components, including drug names and instructions, could be clearly read and interpreted without ambiguity; otherwise, they were classified as illegible.
**WHO AWaRe denominator:** AWaRe percentages were calculated using the total number of antibiotics prescribed as the denominator at each study site, excluding non-antibacterial agents such as antifungals.

### 2.5 Data quality assurance

To ensure data quality and consistency, data collectors were trained before the initiation of the study on standardized procedures for prescription selection, image capture, and data extraction. A structured data extraction format was used to minimize variability in recording information from prescription slips. All extracted data were reviewed for completeness and consistency during data entry. A subset of prescriptions was independently cross-checked by another member of the research team to verify the accuracy of data extraction. Any discrepancies identified during this process were discussed and resolved through consensus. Efforts were also made to ensure clarity in interpreting handwritten prescriptions, and unclear entries were reviewed by senior team members where necessary. These procedures were implemented to enhance the reliability and validity of the collected data.

### 2.6 Procurement and supply data collection

The Bihar Medical Services and Infrastructure Corporation Limited (BMSICL) centrally manages the procurement and distribution of antibiotics in Bihar. It is the sole agency responsible for purchasing, quality-assuring, warehousing, and supplying drugs, equipment, and consumables to all public health facilities under the Department of Health, Government of Bihar.

**Procurement process of drugs through BMSICL**:

1. Estimation of requirements: Annual demand is compiled based on hospital requisitions, Drug Demand Lists (DDLs), and state-level needs.
2. Tendering process: BMSICL issues open, competitive tenders to qualified pharmaceutical manufacturers and suppliers.
3. Evaluation of bids: Suppliers are assessed on quality standards, e.g., GMP (Good Manufacturing Practices) certification, NABL-accredited testing, adherence to product specifications, and cost competitiveness.
4. Finalization and approval: Contracts are awarded to selected suppliers, ensuring transparency and compliance with state procurement rules.
5. Quality assurance: Procured medicines undergo third-party quality testing before release into the supply chain to hospitals and district warehouses.

**Supply process of medicines through BMSICL:**

6. Warehousing: Procured medicines are stored in centralized state warehouses and district depots under controlled conditions.
7. Distribution to hospitals: Drugs are dispatched from these warehouses to government medical colleges, district hospitals, and peripheral health facilities according to requisitions and demand.
8. Hospital-level availability: Clinicians are expected to prescribe from the antibiotics supplied through this centralized system, directly linking procurement and supply with prescribing practices.

For this study, we reviewed BMSICL supply data for the financial years 2022–2025. These records included drug names, year of supply, AWaRe category, and inclusion in India's National Essential Drug List (NEDL). The supply data were then matched with hospital-level prescription patterns to assess alignment between what was prescribed and what was supplied.

### 2.7 Supply–prescription concordance analysis

Antibiotic supply data were obtained from state-level public procurement lists for the financial years 2022–2025. As procurement records did not include quantity-wise, facility-level distribution, stock-out, or dispensing information, antibiotic supply was operationalised as a binary availability variable, coded as “Yes” if an antibiotic appeared in the procurement list for a given year and “No” otherwise. Antibiotics included in supply lists were descriptively compared with antibiotics prescribed in outpatient settings to assess patterns of alignment between supply-side availability and prescribing practices. This analysis was not intended to establish causal relationships.

### 2.8 Data analysis

The data recorded in Excel were imported into a database application for further analysis. Patient demographics, diagnosis categories, and antibiotic use were summarized by number, route, indication, and WHO AWaRe classification, including proportions of empirical, targeted, and prophylactic prescribing, along with other key indicators. Drug supply data were compared with prescribing patterns to assess alignment, stock-outs, and substitutions. Hospital catchment characteristics, including population demographics, rural–urban mix, and referral flows, were compiled from secondary sources and integrated with prescribing and supply data to examine contextual influences on antibiotic use. This analysis was descriptive and exploratory in nature and does not aim to establish causality or independent predictive associations between supply, catchment characteristics, and prescribing patterns. Given the exploratory nature of the study and limitations in data granularity, formal hypothesis testing and inferential statistical modelling were not performed.

## 3 Results

The Results section first describes the demographic and clinical characteristics of outpatient prescriptions across the three tertiary care hospitals, followed by an analysis of prescribing indicators, antimicrobial use patterns, and WHO AWaRe classification. We then examine the alignment between outpatient prescribing and state-level antibiotic supply, interpreted in relation to hospital catchment characteristics.

### 3.1 Demographic characteristics

A total of 8,822 prescriptions were collected over one year from the three tertiary care hospitals in Bihar: Muzaffarpur (3,702), Bhagalpur (1,996), and Darbhanga (3,124). Across all sites, the majority of patients were female (∼56%), and most belonged to the age group 16–39 years (around 46%). The proportion of patients from rural areas was highest at Bhagalpur (98.9%), followed by Darbhanga (82.6%) and Muzaffarpur (71.8%). Most of the patients were reported from the age group 16–39 years (more than 46%) from all three sites. The population that comes to these hospitals is mostly rural, as supported by the data, which is 71% in SKMCH, 82% in DMCH, and 97% in JLNMCH, serving the highest number of patients (Table 2).

**Table 2:** Summarizes key demographics from the three sites.

S. No.	Demographic Variable	Muzaffarpur, *n* (%)	Darbhanga, *n* (%)	Bhagalpur, *n* (%)
1	Gender			
	Male	1,358 (36)	1,383 (44)	868 (43.5)
	Female	2,659 (64)	1,742 (56)	1,128 (56.5)
2	Age group			
	1–5 years	157 (4.3)	212 (6.7)	72 (3.6)
	6–15 years	509 (13.7)	384 (12.3)	195 (9.8)
	16–39 years	1,736 (46.9)	1,505 (48)	1,053 (52)
	40–65 years	1,190 (32.1)	909 (29)	602 (30.2)
	Above 65 years	110 (3)	115 (3.1)	74 (3.7)
3	Owner of the prescription			
	Self	2,521 (68.1)	2,037 (65)	1,240 (62.2)
	Others	1,181 (31.9)	1,087 (35)	756 (37.9)
4	Location of the patient			
	Rural	2,659 (71.8)	2,579 (82.6)	1,974 (97.9)
	Urban	1,043 (28.2)	546 (17.4)	22 (1.1)

According to guidelines by WHO, prescriptions should be made using 100% generic names in all government sectors. From our study, 98% of the prescriptions were made using generic drugs from SKMCH, 99% from both DMCH and JLNMCH. The average number of drugs per prescription was 4.7, 4, and 4 in SKMCH, DMCH, and JLNMCH, respectively. Across the three sites, 100% of prescribed drugs at SKMCH, 84% at DMCH, and 89.4% at JLNMCH were listed in the Essential Drug List (EDL). The remaining prescriptions included antimicrobials not listed in the EDL, indicating that these drugs were not routinely available through the hospital pharmacy system.

A substantial proportion of prescriptions lacked documentation of signs, symptoms, or diagnosis. This was observed in 25.4% (942) of prescriptions at SKMCH, 12% (376) at DMCH, and 7.8% (156) at JLNMCH. Additionally, despite the presence of microbiology laboratories at all three institutions, culture sensitivity testing was advised in only 1.26% (47) of cases at SKMCH, 2.65% (83) at DMCH, and 0.30% (6) at JLNMCH. The key prescribing indicators across the three hospitals are summarized in Figure 1.

### 3.2 Commonly prescribed antimicrobials

**Figure 2:**
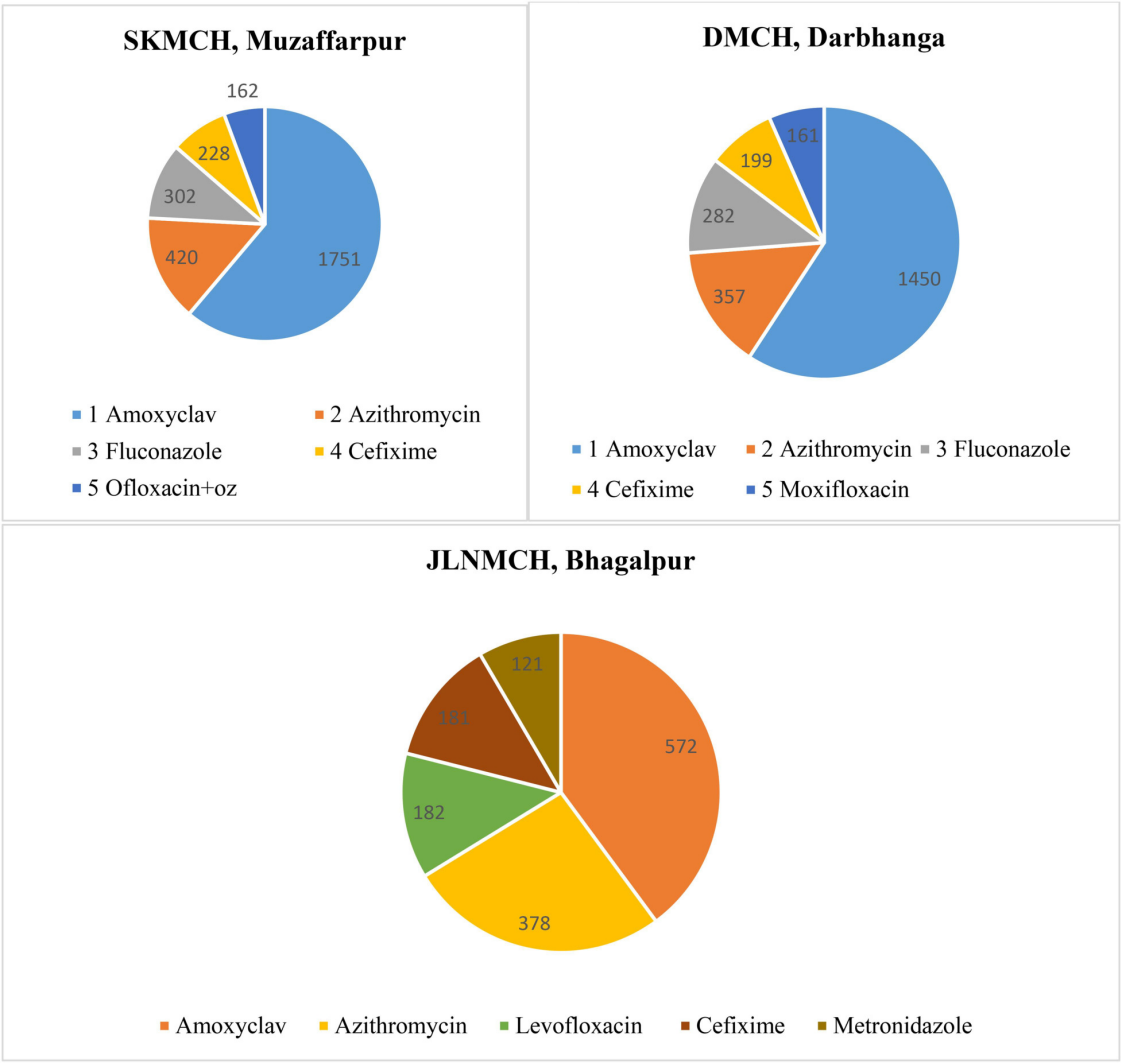
Number of the top five commonly prescribed antimicrobials in all three sites.

The most frequently prescribed antimicrobials across the three sites were Amoxicillin–Clavulanic acid, followed by Azithromycin and Cefixime, which were common across all hospitals. Site-specific variations were observed: Fluconazole and Ofloxacin–Ornidazole were among the top prescribed agents at SKMCH, while Fluconazole and Moxifloxacin were more prominent at DMCH. In contrast, Levofloxacin and Metronidazole appeared among the most frequently prescribed antimicrobials at JLNMCH. These patterns are consistent with the antimicrobial distribution presented in Figure 2.

### 3.3 OPD wise distribution

**Figure 3:**
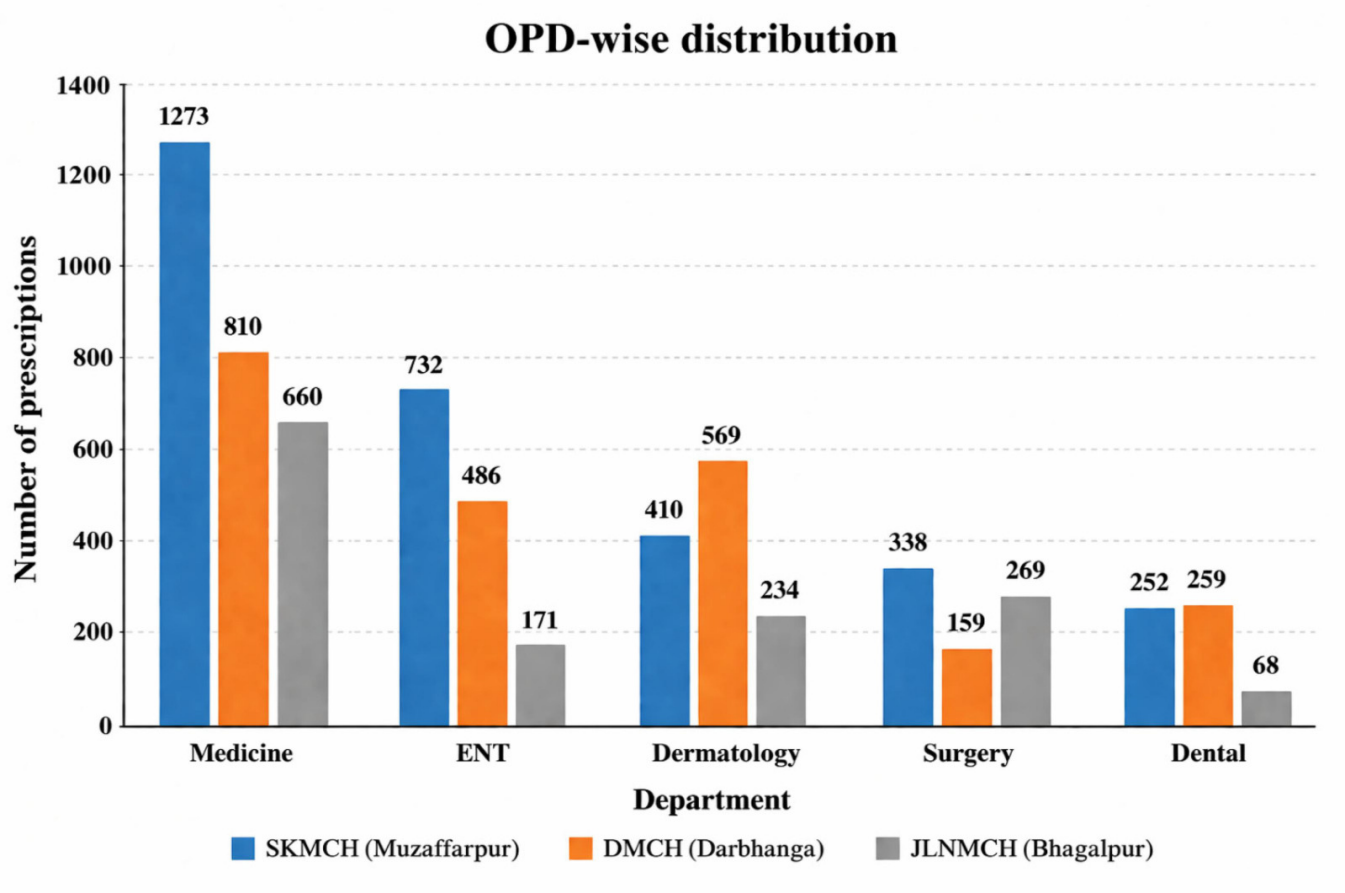
OPD-wise distribution of prescriptions.

The prescription was made most commonly from the Medicine OPD, followed by ENT, Dermatology, Surgery, and Dental OPD (Figure 3).

### 3.4 Signs and symptoms

**Figure 4:**
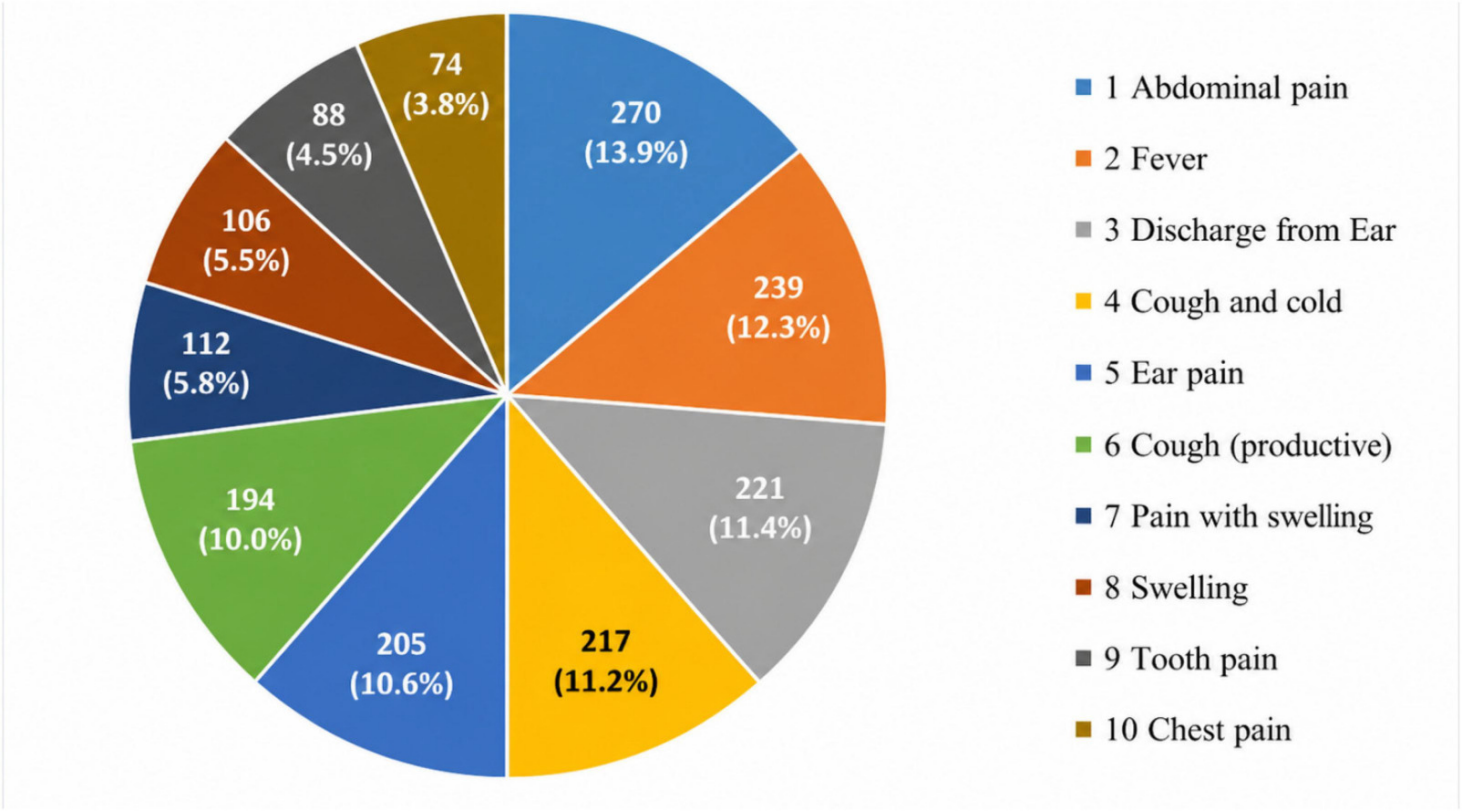
Distribution of top 10 reported symptoms Among OPD patients.

Top signs and symptoms reported from all three sites were: abdominal pain, fever, Discharge from the ear, cough and cold, ear pain, swelling, tooth pain, and chest pain. For these conditions, antimicrobials were prescribed (Figure 4).

#### 3.4.1 WHO core indicator analysis

**Figure 5:**
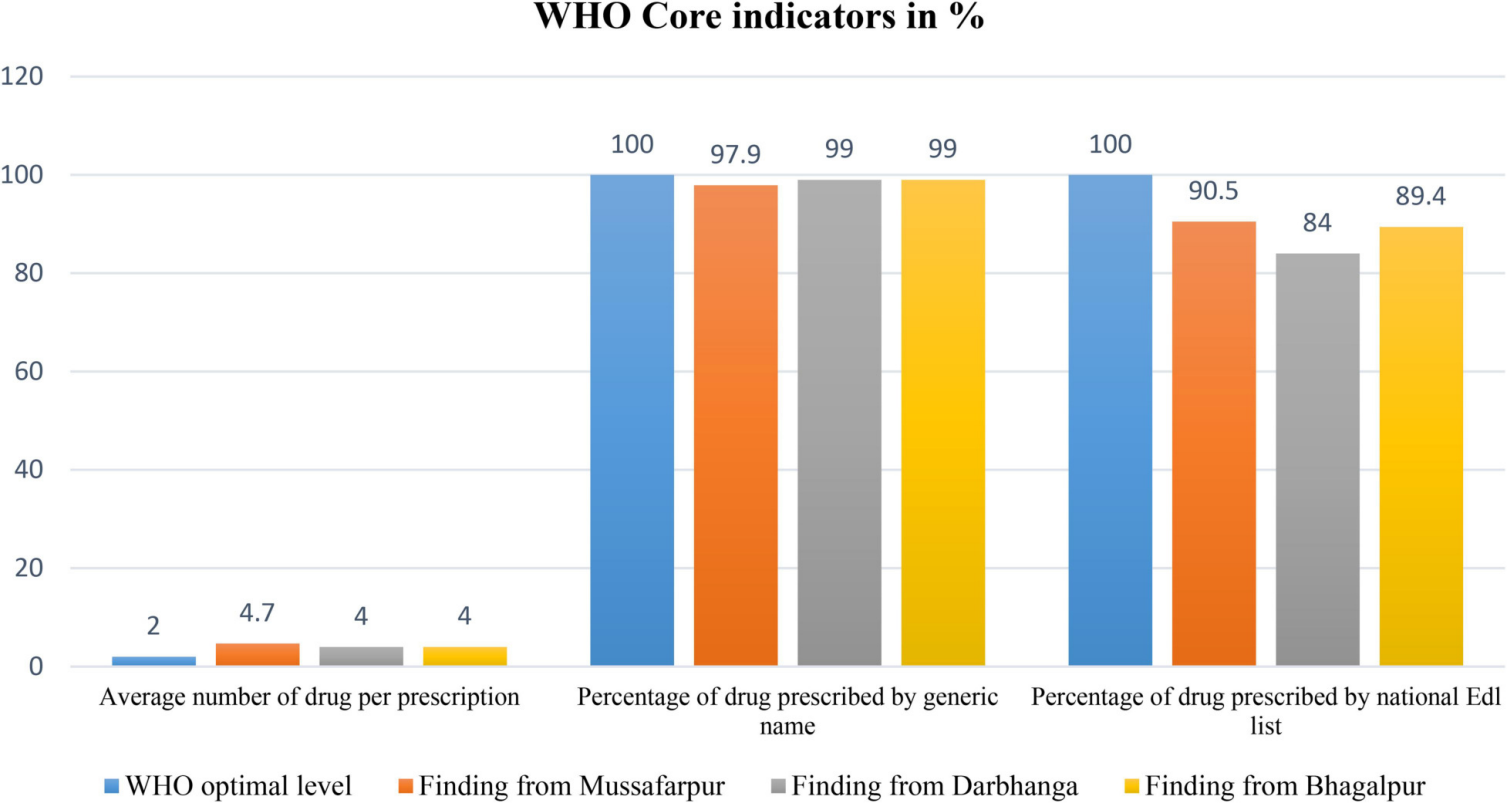
WHO core prescribing indicators vs. findings from Muzaffarpur, Darbhanga, and Bhagalpur.

WHO prescribing indicators were also studied in these three settings (Figure 5). The 3 main indicators studied were:

- An average number of drugs prescribed per encounter.
- Percentage of drugs prescribed by generic name.
- Percentage of drugs prescribed from the National Essential Drug List (NEDL).

### 3.5 WHO AWaRe classification

**Figure 6:**
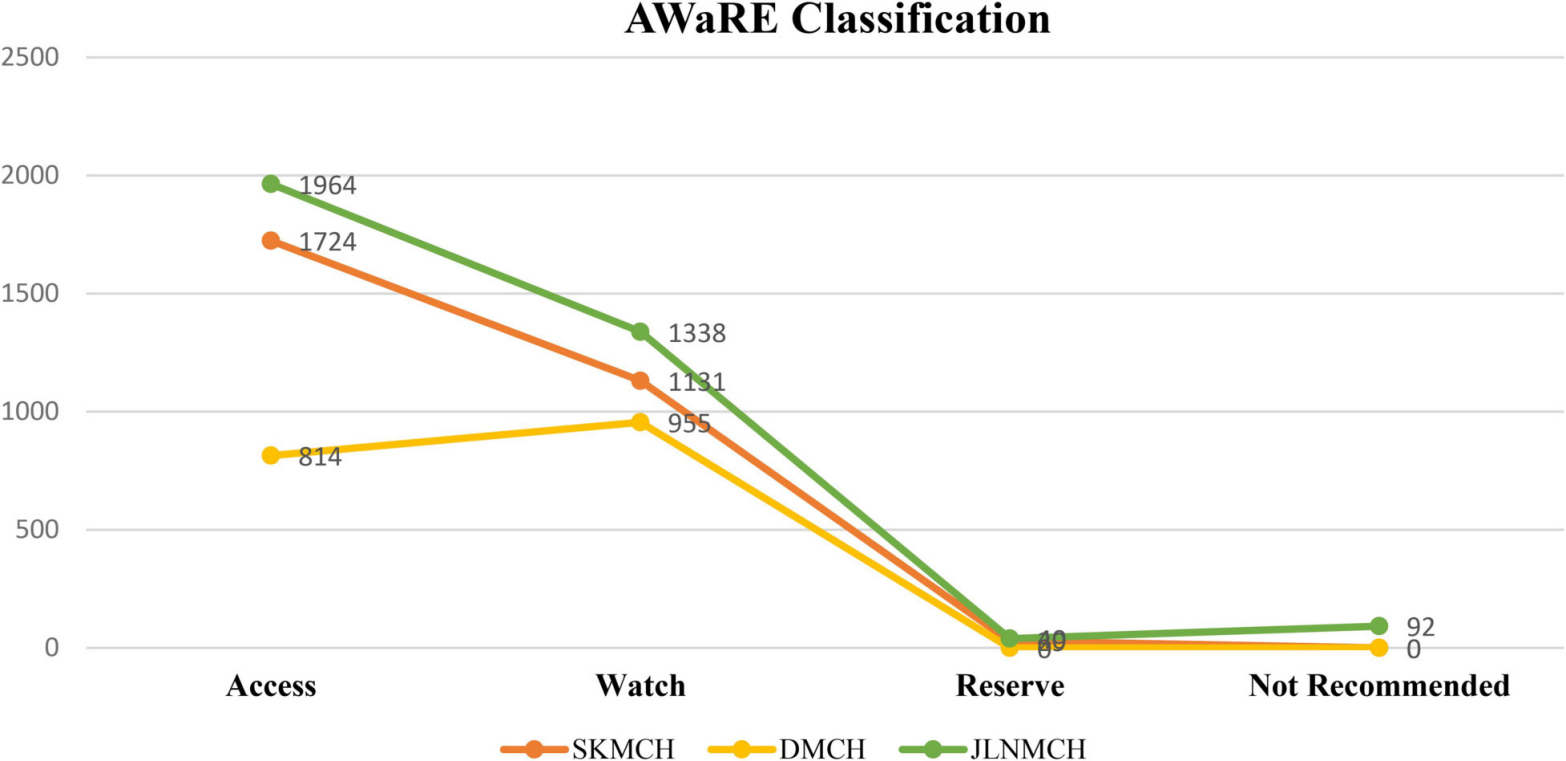
Comparative overview of WHO AWaRe-based antibiotic prescriptions across the three sites.

AWaRe percentages were calculated based on the total number of antibiotics prescribed at each site, excluding antifungal agents. As per the WHO AWaRe classification, from SKMCH Muzaffarpur 54% (1,964) of the antimicrobials were prescribed from the Access category followed by 36% (1,338) from the Watch and only 2% (92) from the not recommended group and none from the reserve group whereas from DMCH, 55.2% (1,724) were from the Access category and 35.80% (1,131) from the Watch category. There was no antimicrobial from the reserve and the not-recommended group. From JLNMCH, 58% (955) were from the Watch category and 42% (814) from the Access category. No antimicrobials were prescribed from the reserve and the not-recommended group (Figure 6).

### 3.6 Alignment between antibiotic supply, outpatient prescribing patterns, and catchment population

Table 3 compares the eight most frequently prescribed antibiotics across the three study sites with their supply data (antibiotics dispatched by BMSICL to hospitals) for the period 2022–2025, along with AWaRe classification, NEDL inclusion, and key stewardship considerations. In our findings, Amoxicillin–clavulanic acid was the most frequently prescribed antibiotic overall, representing 42.8% of all prescriptions, with particularly high use at JLNMCH (72.6%). This antibiotic was supplied consistently over the three years, is classified under the Access category, and is included in the NEDL, indicating an observed alignment between clinical demand and supply priorities. Similar alignment was observed for Azithromycin (13.1% of prescriptions) and Cefixime (6.9%), both of which were regularly supplied, listed in the NEDL, and classified under the Watch category. Fluconazole, prescribed in 6.6% of cases overall, was also consistently supplied, despite not being classified within the AWaRe framework due to its antifungal status. Levofloxacin (2.1%) and Metronidazole (1.4%) demonstrated supply–prescription concordance at the facilities where they were used. However, notable mismatches were identified. Moxifloxacin, a Watch category agent not included in the NEDL, was prescribed at JLNMCH (8.1%) and supplied by BMSICL, raising concerns regarding the distribution of non-essential broad-spectrum agents. Furthermore, Ofloxacin–Ornidazole, an irrational fixed-dose combination not listed in the NEDL or WHO recommendations, was prescribed at SKMCH (4.4%) but not supplied, implying patients likely obtained it outside the hospital system.

**Table 3:** Comparison of prescription patterns and supply alignment for Top antibiotics across three tertiary care hospitals in Bihar (2023–2024).

Antibiotic	Antimicrobial Prescription Data				Antimicrobial Suppjjly Data				
	SKMCH (Muzaffarpur)	DMCH (Darbhanga)	JLNMCH (Bhagalpur)	Total Prescribed *n* (%)	Supplied by BMS CL	AWaRe Category	EDL Status	Years	Comments
Amoxyclav	1,751 (47.3%)	572 (18.3%)	1,450 (72.6%)	3,773 (42.8%)	yes	Access	Yes	2022–2025	Most prescribed and supplied
Azithromycin	420 (11.3%)	378 (12.1%)	357 (17.9%)	1,155 (13.1%)	Yes	Watch	Yes	2022–2025	Most supplied
Cefixime	228 (6.2%)	181 (5.8%)	199 (10.0%)	608 (6.9%)	Yes	Watch	Yes	2022–2025	Aligned
Fluconazole	302 (8.2%)	0 (0.0%)	282 (14.1%)	584 (6.6%)	Yes	Not classified (antifungal)	Yes	2022–2025	Aligned
Levofloxacin	0 (0.0%)	182 (5.8%)	0 (0.0%)	182 (2.1%)	Yes	Watch	Yes	2022–2025	Aligned
Metronidazole	121 (3.3%)	0 (0.0%)	0 (0.0%)	121 (1.4%)	Yes	Access	Yes	2022–2025	Aligned
Moxifloxacin	0 (0.0%)	0 (0.0%)	161 (8.1%)	161 (1.8%)	Yes	Watch	No	2022–2025	not aligned
Ofloxacin + Ornidazole	162 (4.4%)	0 (0.0%)	0 (0.0%)	162 (1.8%)	No	Watch	No	Not procured	not on EDL

Table 4 presents a comprehensive summary of catchment characteristics, hospital infrastructure, prescription indicators, Outpatient Department-Wise distribution, and the most commonly prescribed antimicrobials across the three study sites: SKMCH, DMCH, and JLNMCH in Bihar. Across all sites, the majority of patients were from rural areas (72%–98%), reflecting the hospitals' extensive rural catchments. Despite comparable bed strength and high OPD loads (∼2,000–3,000 patients per day), notable differences were observed in prescription practices. The average number of drugs per prescription was consistently high (4–4.7), but culture test advice remained very low (0.3%–2.6%), indicating heavy reliance on empirical prescribing. Documentation quality varied: SKMCH had the highest proportion of prescriptions without a recorded diagnosis (25.4%), whereas DMCH showed better documentation of illness duration (28%). Medicine OPDs were the predominant source of antibiotic prescriptions at all sites, followed by ENT and dermatology. The top antimicrobials prescribed were broad-spectrum agents, with Amoxicillin–Clavulanic acid, Azithromycin, and Cefixime ranking highest across all hospitals. Importantly, the AWaRe analysis revealed that while Access-category antibiotic use was above 50% at SKMCH and DMCH, JLNMCH showed a concerning 58% of prescriptions from the Watch category.

**Table 4:** Comparative summary of catchment characteristics, hospital infrastructure, prescription indicators, OPD distribution, and top antimicrobials across SKMCH, DMCH, and JLNMCH in Bihar.

Indicator	SKMCH (Muzaffarpur)	DMCH (Darbhanga)	JLNMCH (Bhagalpur)
Number of prescriptions analyzed	3,702	3,124	1,996
Catchment type	Semi-urban + rural; 102 PHCs, 17 CHCs; largest referral center	Semi-urban + rural; 90 PHCs, 15 CHCs; old referral center	Urban + extensive rural; 65 PHCs, 11 CHCs; sparse rural clinics
Bed strength/OPD load	∼900 beds; ∼2,500–3,000/day OPD	∼800 beds; ∼2,000–2,500/day OPD	∼750 beds; ∼2,000/day OPD
% Rural patients	71.8%	82.6%	97.9%
Average drugs/prescription	4.7	4	4
% Culture test advice	1.26% (47 cases)	2.65% (83 cases)	0.3% (6 cases)
% Multiple antibiotics	32.7% (1,213 cases)	39.3% (1,230 cases)	22.8% (456 cases)
% No diagnosis mentioned	25.4% (942 cases)	12% (376 cases)	7.8% (156 cases)
% Illness duration recorded	12.07% (447 cases)	28% (901 cases)	18.8% (375 cases)
% Legible prescriptions	80.8% (2,994 cases)	87.4% (2,730 cases)	98% (1,955 cases)
% Generic prescribing	97.9% (3,627 cases)	99% (3,091 cases)	99% (1,975 cases)
% Access antibiotics (AWaRe)	53% (1,964 cases)	55.2% (1,724 cases)	41% (814 cases)
% Watch antibiotics (AWaRe)	36% (1,338 cases)	36% (1,131 cases)	58% (955 cases)
Top prescribing OPDs	1. Medicine, 2. ENT, 3. Dermatology, 4. Surgery, 5. Dental	1. Medicine, 2. ENT, 3. Dermatology, 4. Surgery, 5. Dental	1. Medicine, 2. ENT, 3. Dermatology, 4. Surgery, 5. Dental
Top 5 antimicrobials (n)	1. Amoxyclav (1,751), 2. Azithromycin (420), 3. Fluconazole (302), 4. Cefixime (228), 5. Oflox–Ornidazole (162)	1. Amoxyclav (1,450), 2. Azithromycin (357), 3. Fluconazole (282), 4. Cefixime (199), 5. Moxifloxacin (161)	1. Amoxyclav (572), 2. Azithromycin (378), 3. Levofloxacin (182), 4. Cefixime (181), 5. Metronidazole (121)

## 4 Discussion

This multicenter study provides the first comprehensive evidence on outpatient antibiotic prescribing in Bihar, showing how supply practices and catchment dynamics influence use and offering critical guidance for stewardship in resource-limited settings. By triangulating the antibiotics prescribed, the antibiotics consistently supplied through centralized procurement, and the population contexts in which tertiary hospitals operate, the study moves beyond prescriber-centric explanations to a health-systems understanding of antibiotic use. However, this study is exploratory and descriptive in nature and does not aim to establish causality or independent predictive relationships between supply stability, catchment characteristics, and prescribing patterns. In our findings, the most commonly prescribed antimicrobials from the three sites were Amoxicillin + Clavulanic acid, followed by Azithromycin and Cefixime. These three agents all belong to the broad-spectrum category. All three were also among the most consistently supplied antibiotics by BMSICL during 2022–2025; the consistent availability of these antibiotics may be associated with their continued dominance in prescribing patterns. This overlap indicates that stable and repeated inclusion of specific antibiotics within the state supply lists plays a key role in reinforcing their continued dominance in outpatient prescribing.

A prescription study by Mugada et al. (9) similarly reported the same antibiotics as the most commonly prescribed. This suggests that the heavy reliance on broad-spectrum agents is not site-specific but reflects a wider trend in tertiary care settings (9). This study also supports the results of Kaur et al. (14), who found that greater than 65% of prescriptions were from broad-spectrum antimicrobials such as Amoxicillin-Clavulanic acid, Ceftriaxone, Ciprofloxacin, Clindamycin, and Piperacillin-tazobactam (14). This shows that prescribers usually rely on broad-spectrum antibiotics rather than narrow-spectrum ones. Importantly, in our study, the extent of broad-spectrum prescribing varied notably across sites: approximately 36% at SKMCH and DMCH, and as high as 58% at JLNMCH. These differences are best understood in relation to hospital catchment characteristics rather than supply differences, as procurement is centralized and uniform across the state. The predominance of broad-spectrum use at JLNMCH may reflect contextual factors related to its rural-extensive catchment. It is plausible that longer patient travel times, limited opportunities for follow-up, and constraints in diagnostic access could influence clinicians to prefer broader empirical treatments. Catchment characteristics derived from secondary sources are used here to contextualize the observed prescribing patterns rather than to establish direct explanatory relationships. However, these interpretations are not directly tested in the present study and should be considered as hypotheses for future investigation. This temporal stability in supply may have entrenched prescribing habits, as doctors continued to rely on the same broad-spectrum agents readily available through the supply chain.

Our findings highlight a high number of female patients, implying that more females were prescribed antibiotics than male patients, which was similar to a study conducted in Himachal Pradesh, where females accounted for 56% of the total prescriptions made (15). This pattern may also be influenced by the catchment demographics, as all three hospitals cater predominantly to rural populations where women often seek care for reproductive, maternal, and infectious conditions, contributing to higher female outpatient attendance and antibiotic prescribing. Further, we found the average number of drugs per prescription to be 4.7, 4, and 4 at SKMCH, DMCH, and JLNMCH, respectively, significantly higher than the WHO recommendation of 1.6–1.8 drugs per encounter (16). This high level of polypharmacy reflects the pressures of tertiary outpatient care in Bihar, where hospitals serve large rural catchments with limited access to primary care, leading patients to seek comprehensive treatment in a single visit. A study from Himachal Pradesh reported an average of 2.6 drugs per prescription, which is comparatively lower (15). This means that, on average, 4–5 drugs are prescribed per outpatient visit in our Bihar sites, indicating a substantial level of polypharmacy. One likely reason for this is the specific healthcare context of Bihar, where large rural catchments, limited access to primary care, and long patient travel times result in patients seeking “complete treatment” in a single hospital visit. Clinicians, facing overwhelming OPD loads and limited follow-up opportunities, may prescribe multiple medications to meet patient expectations and reduce the perceived need for return visits (17). Additionally, weak referral systems and inadequate diagnostic capacity may further drive symptom-based prescribing, increasing the total number of drugs per encounter. Over the three-year supply period, the list of most supplied antibiotics remained largely the same, with Amoxicillin + Clavulanic acid, Azithromycin, and Cefixime consistently supplied across all sites. This temporal stability in supply patterns may reinforce entrenched prescribing behaviours, particularly when Watch group antibiotics such as Azithromycin and Cefixime remain readily available year after year.

A culture sensitivity test is performed to determine the antimicrobial susceptibility of disease-causing microorganisms. However, in our study, culture tests were advised in only 1.26% (47) of the total 3,702 cases at SKMCH, 2.65% at DMCH, and a mere 0.3% at JLNMCH. This is concerning, especially given that all three are tertiary medical colleges with well-established microbiology departments and access to nearby private laboratories. This low reliance on diagnostic confirmation highlights a critical disconnect between laboratory capacity and routine outpatient workflows, particularly in settings serving rural catchments where patients may be unable to return for follow-up once results become available. This finding highlights a critical gap in stewardship and reflects the reliance on empirical therapy without culture sensitivity testing in tertiary care settings. For instance, Modgil et al. (18) reported that only 2% of patients with suspected infections underwent culture testing in a tertiary care hospital in India, despite widespread use of broad-spectrum antibiotics (18). Likewise, Chandy et al. (19) found that less than 10% of antibiotic prescriptions were guided by laboratory results (19). In many hospitals, especially in LMICs, antibiotics are often prescribed empirically, meaning the patient is given antibiotic therapy without laboratory confirmation of the causal organism. The reasons behind this include limited rapid diagnostic tools, time constraints due to delayed results (often taking 48–72 h), limited laboratory infrastructure, financial barriers for patients, and habitual reliance on empirical prescribing (20). In the specific context of Bihar, the low culture testing at tertiary hospitals can be explained by its extensive rural catchment, where patients often travel more than 4 h to reach the hospital and have limited ability to return for follow-up visits, pushing doctors to prioritize immediate empirical treatment over waiting for lab confirmations. Even at SKMCH and DMCH, slightly higher (but still low) culture testing rates may reflect their semi-urban locations and better lab access, yet they remain constrained by overwhelming OPD volumes, insufficient diagnostic integration, and a health system not structured to support routine microbiological confirmation in outpatient care.

In our study, more than 97% of prescriptions from all three sites were given using the generic name. This practice reflects institutional norms within public sector hospitals and aligns with the socioeconomic realities of the hospitals' catchment populations, which are predominantly rural and low-income. This prescribing practice was far better than those reported by Green et al. (88%) (21). Prescribing drugs by their generic names is essential in LMICs because it lessens the economic burden on poor people. Patients' misconceptions about generic drugs vs. brand drugs allow easy exploitation and make them prefer branded drugs over generic drugs. Moreover, prescribing generic drugs often prevents confusion surrounding multiple names assigned to the same product. Patients are also habituated to buy the drug with the same brand name only, despite its availability in generic form or under another brand. There is a need to strengthen the awareness of generic drugs and their availability among patients. In India, the central government has set up a “Jan Aushadhi” scheme wherein pharmacies will sell generic drugs and all medicines so that pharmacies are affordable for common people (22). Our study suggests that the consistently high rate of generic prescribing across the three hospitals is not only a reflection of good institutional practices but also closely linked to the socioeconomic realities of their catchment populations. The majority of patients served come from rural, low-income backgrounds with limited capacity to afford branded medicines, making generic prescribing both a practical and necessary approach to ensure treatment access. In Bihar, where government hospitals often act as the main source of affordable care across vast rural regions, maintaining strong generic prescribing practices is critical to reducing out-of-pocket expenses and protecting household-level financial security.

Another concerning observation from our study was the prescription of antimicrobials in cases where no signs, symptoms, or diagnosis were recorded on the prescription slips to justify their use. This pattern of apparently indiscriminate prescribing is consistent with findings from previous studies in India and other LMICs; Kotwani and Holloway (23) reported that a significant proportion of antibiotic prescriptions in Delhi were issued without any documented infection-related diagnosis (23), and Kumar et al. (24) similarly observed empirical antibiotic use even in the absence of clear clinical indications (24). Such practices not only expose patients to unnecessary adverse effects but also accelerate the emergence of AMR (25). Notably, SKMCH had the highest percentage (25.4%) of prescriptions without documented diagnosis, which may reflect extreme OPD crowding at this largest referral center in Bihar, where doctors are under intense pressure to handle large patient loads with minimal time per consultation. JLNMCH and DMCH fared slightly better, suggesting that documentation quality is shaped not just by individual prescriber behaviour but also by patient volume and catchment pressures. This variation in documentation quality appears closely linked to differences in outpatient volume and referral burden, with the largest referral center experiencing the greatest documentation gaps.

Our supply–prescription analysis revealed both alignments and mismatches. On the positive side, Amoxicillin–Clavulanic acid, Azithromycin, and Cefixime were the most frequently prescribed antibiotics and were also consistently supplied by BMSICL across multiple years, ensuring availability and minimizing stock-outs. However, the continued supply of Watch-category and non-EDL antibiotics such as Moxifloxacin raises concerns about how procurement decisions may contribute to the continued availability of antibiotics that are frequently prescribed. Rather than reflecting volume-based availability, this alignment highlights the stability and persistence of specific antibiotics within the state supply lists, which may reinforce entrenched prescribing practices over time. At the same time, the role of BMSICL in strengthening Bihar's public health supply chain is noteworthy: centralized procurement and distribution have improved transparency, reduced costs, enhanced quality assurance, and ensured more uniform drug availability across facilities. These systemic gains create a strong foundation for stewardship if supply decisions are more closely aligned with AWaRe and NEDL priorities. At JLNMCH, the higher reliance on Watch-category antibiotics appears to reflect the interaction between stable availability of broad-spectrum agents and the hospital's predominantly rural catchment, where clinicians may favor empirical “stronger” therapy due to limited diagnostic access and reduced likelihood of patient follow-up. This highlights the need to tailor stewardship strategies to local catchment realities while aligning supply priorities with rational use frameworks such as AWaRe. This stability reinforces the point that entrenched prescribing behaviors are perpetuated by consistent procurement of the same antibiotics year after year. At the same time, the close overlap between forecast and supply values also highlights the limited scope for diversifying the antibiotic mix available at these facilities, with Watch group antibiotics such as Azithromycin and Cefixime remaining reliably stocked. While this alignment reduces the risk of stock-outs, it may inadvertently sustain patterns of broad-spectrum prescribing, especially in high-volume outpatient settings.

Antimicrobial stewardship is crucial to combating AMR, and the WHO AWaRe classification provides a globally endorsed framework to monitor and optimize antibiotic use (25). In the present study, a significant proportion of prescriptions were from the Access category at SKMCH (54%) and DMCH (55.2%), aligning with the WHO recommendation that at least 60% of antibiotic consumption should come from Access group antibiotics (26), reflecting relatively rational use in these centers. However, JLNMCH showed a higher proportion of Watch group prescriptions (58%), exceeding the recommended thresholds and suggesting potential overuse of broad-spectrum agents, which raises concerns about promoting resistance (27–29). Gandra and Kotwani (30) similarly emphasized the need to improve the availability of Access group antibiotics and reduce the use of Watch group antibiotics in India to optimize antibiotic use and contain AMR. The persistence of these patterns is likely reinforced by the stable availability of Watch group antibiotics in the supply lists across 2022–2025, underlining the need for supply policies that actively prioritize Access category agents. The higher Watch use at tertiary care may be partly explained by its remote, predominantly rural catchment area and lack of lower-level facilities, where providers may opt for broad-spectrum antibiotics as a “safety net” when follow-up is unlikely, in contrast to DMCH's more balanced Access-to-Watch prescribing, likely aided by its semi-urban referrals and slightly better infrastructure. Overall, these site-level differences highlight how healthcare infrastructure, patient demographics, referral patterns, and catchment pressures directly shape antimicrobial prescribing practices, diagnostic reliance, and stewardship opportunities, underscoring the need for interventions that are tailored to local health system realities rather than one-size-fits-all solutions.

Overall, these findings highlight that prescribing behaviours in Bihar's public hospitals are shaped not only by patient demographics and catchment realities but also by the antibiotics that supply systems make available year after year. Although antibiotic procurement decisions are centralized at the state level, their effects are experienced differently at individual hospitals depending on catchment characteristics such as rurality, patient volume, travel time, and diagnostic access. Prescribing behavior thus emerges at the intersection of stable supply availability and local healthcare realities. This triangulation of prescription data, supply patterns, and catchment context provides a more complete explanation for persistent reliance on broad-spectrum and empirical antibiotic use than any single factor alone. These contextual interpretations are intended to generate hypotheses and highlight potential health-system dynamics rather than establish causal explanations.

### 4.1 Limitations

This study has several limitations. Prescription sampling was restricted to a fixed high-volume OPD time window (10:00 AM to 1:00 PM), which may introduce selection bias and not capture full-day or seasonal variation. Antibiotic supply data were derived from state-level procurement lists and operationalised as binary availability indicators, lacking information on quantities, facility-level distribution, stock-outs, and dispensing practices; thus, supply–prescription alignment should be interpreted as descriptive rather than causal. The absence of facility-level pharmacy data further limits insight into point-of-care availability. The analysis was exploratory and did not include inferential statistical modelling. Catchment characteristics were based on secondary sources such as the (11) and are used for contextual interpretation only, as they may not reflect current conditions. Indications for antibiotic use were based on prescription records, rather than clinical validation, which introduced potential misclassification. Finally, findings from three public tertiary hospitals in Bihar may not be generalizable to other settings. Future studies using multivariable approaches and more granular facility-level data are needed to better understand system-level drivers of prescribing behaviour.

### 4.2 Implications for antimicrobial stewardship policies

Our study highlights critical gaps in antimicrobial prescribing practices across public tertiary care hospitals in Bihar, with implications for stewardship policies. Given below are the key points:

- There is high use of broad-spectrum (Watch group) antibiotics, underscoring the need to promote Access group antibiotics as per WHO AWaRe targets.
- Minimal use of culture sensitivity testing indicates the urgent need to strengthen microbiology services, introduce rapid diagnostics, and better integrate lab data into prescribing decisions.
- High polypharmacy rates reflect clinician pressures in overcrowded OPDs and call for focused training on rational prescribing under real-world conditions.
- Poor documentation practices, including prescriptions without recorded diagnoses, highlight the need to improve prescription writing standards and promote accountability.
- Encouragingly, the consistently high rate of generic prescribing reflects positive institutional norms and should be maintained to ensure affordable access, particularly for low-income, rural patients.
- Overall, stewardship interventions must be context-sensitive, addressing local barriers such as patient travel challenges, weak referral systems, and infrastructure constraints, rather than applying one-size-fits-all solutions.

## 5 Conclusion

These findings highlight that antibiotic use in Bihar's public hospitals is shaped not only by prescriber choice but also by the sustained availability of specific antibiotics within centralized procurement/supply systems. Our findings reveal widespread reliance on broad-spectrum (Watch group) antibiotics, high levels of polypharmacy, limited use of culture testing, and gaps in prescription documentation, challenges most acute in rural, high-volume centers. At the same time, the near-universal use of generic names demonstrates positive prescribing practices that promote affordability and access for low-income patients. Taken together, these results highlight that antibiotic use is shaped not only by prescriber choice but also by systemic drivers, including supply stability, diagnostic constraints, and catchment pressures. Tackling AMR in Bihar and comparable low-resource settings, therefore, demands context-sensitive stewardship strategies that explicitly link supply-chain reform, diagnostic integration, and prescriber support rather than relying on uniform, top-down approaches.

## Data Availability

The raw data supporting the conclusions of this article will be made available by the authors, without undue reservation.

## References

[1] Sulis G Adam P Nafade V Gore G Daniels B Daftary A. Antibiotic prescription practices in primary care in low- and middle-income countries: a systematic review and meta-analysis. PLoS Med. (2020) 17:e1003139. 10.1371/journal.pmed.1003139 32544153 PMC7297306

[2] Saleem Z Mekonnen BA Orubu ES Islam MA Nguyen TTP Ubaka CM. Current access, availability and use of antibiotics in primary care among key low- and middle-income countries and the policy implications. Expert Rev Anti Infect Ther. (2025) 23:1–42. 10.1080/14787210.2025.2477198 40110804

[3] Salam MA Al-Amin MY Salam MT Pawar JS Akhter N Rabaan AA. Antimicrobial resistance: a growing serious threat for global public health. Healthcare (Basel). (2023) 11:1946. 10.3390/healthcare11131946 37444780 PMC10340576

[4] Francis J Abraham S. Clinical pharmacists: bridging the gap between patients and physicians. Saudi Pharm J. (2014) 22:600–2. 10.1016/j.jsps.2014.02.011 25561874 PMC4281611

[5] Modgil V Sahay S Taneja N Qayyumi B Singh R Mukherjee A. Mapping the AMR infection landscape in Bihar: implications for strengthening policy and clinical practice. Antibiotics. (2025) 14:684. 10.3390/antibiotics14070684 40723987 PMC12291626

[6] Idris SA Hussien TMA Al-Shammari FF Nagi HA Bashir AI Elhussein GEMO. An evaluation of drug prescribing patterns and prescription completeness. Healthcare (Basel). (2024) 12:2221. 10.3390/healthcare12222221 39595419 PMC11594192

[7] Modgil V Shafiq N Gondara A Surial R Singh H Karol V. An evaluation of antibiotic prescription pattern and drug rationality analysis among outpatients at public health setting, India. Indian J Med Microbiol. (2025) 55:100829. 10.1016/j.ijmmb.2025.100829 40157426

[8] Sharma A Singh A Dar MA Kaur RJ Charan J Iskandar K. Menace of antimicrobial resistance in LMICs: current surveillance practices and control measures to tackle hostility. J Infect Public Health. (2022) 15:172–81. 10.1016/j.jiph.2021.12.008 34972026

[9] Mugada V Mahato V Andhavaram D Vajhala SM. Evaluation of prescribing patterns of antibiotics using selected indicators for antimicrobial use in hospitals and the access, watch, reserve (AWaRe) classification by the world health organization. Turk J Pharm Sci. (2021) 18:282–8. 10.4274/tjps.galenos.2020.11456 34157817 PMC8231325

[10] Fazaludeen Koya S Ganesh S Selvaraj S Wirtz VJ Galea S Rockers PC. Antibiotic consumption in India: geographical variations and temporal changes between 2011 and 2019. JAC Antimicrob Resist. (2022) 4:dlac112. 10.1093/jacamr/dlac112 36320447 PMC9596537

[11] Census of India. Primary Census Abstract: Bihar. New Delhi: Registrar General and Census Commissioner, Government of India (2011).

[12] Government of Bihar. District Health Portals—Bihar. Patna: Health Department, Government of Bihar (n.d.).

[13] National Health Mission. Bihar State Health Profile. New Delhi: Ministry of Health and Family Welfare, Government of India (2023).

[14] Kaur A Bhagat R Kaur N Shafiq N. A study of antibiotic prescription pattern in patients referred to tertiary care center in northern India. Ther Adv Infect Dis. (2018) 5(4):63–8. 10.1177/2049936118773216 30013773 PMC6044109

[15] Mukherjee A Surial R Sahay S Thakral Y Gondara A. Social and cultural determinants of antibiotics prescriptions: analysis from a public community health centre in north India. Front Pharmacol. (2024) 15:1277628. 10.3389/fphar.2024.1277628 38333004 PMC10850286

[16] Meenakshi R Selvaraj N Anandabaskar N Dhamodharan A Badrinath AK Rajamohammad MA. Prescription audit of a teaching hospital in south India using world health organization core prescribing indicators—a cross-sectional study. Perspect Clin Res. (2022) 13:132–6. 10.4103/picr.PICR_172_20 35928646 PMC9345256

[17] Nasehi MM Effatpanah M Gholamnezhad M Karami H Ghamkhar M Armand N. Antibiotic prescription prevalence in Iranian outpatients: a focus on defined daily doses and the AWaRe classification system. Am J Infect Control. (2024) 52(12):1359–65. 10.1016/j.ajic.2024.07.007 39032834

[18] Modgil V Sahay S Mukherjee A Banta R Joshi N Surial R. Integrating procurement, prescription, and resistance data to strengthen antimicrobial stewardship: insights from a public health institution in India. Front Microbiol. (2025) 16:1673019. 10.3389/fmicb.2025.1673019 41244685 PMC12615211

[19] Chandy SJ Naik GS Balachander J Jeyaseelan L Thomas K Stalsby Lundborg C. The impact of policy guidelines on hospital antibiotic use over a decade: a segmented time series analysis. PLoS One. (2013) 8:e55499. 10.1371/journal.pone.0055499 24647339 PMC3960230

[20] World Health Organization. Antimicrobial Resistance: Global Report on Surveillance 2014. Geneva: WHO (2014).

[21] Green DL Keenan K Fredricks KJ Huque SI Mushi MF Kansiime C. The role of multidimensional poverty in antibiotic misuse: a mixed-methods study of self-medication and non-adherence in Kenya, Tanzania, and Uganda. Lancet Glob Health. (2023) 11:e59–68. 10.1016/S2214-109X(22)00423-5 36521953

[22] Rocke T El Omeiri N Quiros RE Hsieh J Ramon-Pardo P. Reporting on antibiotic use patterns using the WHO access, watch, reserve classification in the Caribbean. Rev Panam Salud Publica. (2022) 46:e186. 10.26633/RPSP.2022.186 36382253 PMC9642817

[23] Kotwani A Holloway K. Trends in antibiotic use among outpatients in New Delhi, India. BMC Infect Dis. (2011) 11:99. 10.1186/1471-2334-11-99 21507212 PMC3097160

[24] Kumar R Singh J Meena GS Jain SK. Antibiotic prescription practices in primary health care settings in India: a mixed-methods study. BMC Public Health. (2019) 19:1–9. 10.1186/s12889-019-7292-330606151

[25] World Health Organization. Global Action Plan on Antimicrobial Resistance. Geneva: WHO (2015). 10.7196/samj.9644 26242647

[26] Klein EY Milkowska-Shibata M Tseng KK Sharland M Gandra S Pulcini C. Assessment of WHO antibiotic consumption and access targets in 76 countries, 2000–15: an analysis of pharmaceutical sales data. Lancet Infect Dis. (2021) 21:107–15. 10.1016/S1473-3099(20)30332-7 32717205

[27] Vijay S Ramasubramanian V Bansal N Ohri VC Walia K. Hospital-based antimicrobial stewardship, India. Bull World Health Organ. (2023) 101:20–7A. 10.2471/BLT.22.288797 36593779 PMC9795386

[28] Abdou E Hayder R Salaheldin M Eltayeb E. Over-prescription of watch antibiotics in primary healthcare settings in Sudan: results from routinely collected prescription data. J Infect Dev Ctries. (2025) 19(01):91–7. 10.3855/jidc.20106 39977472

[29] Aarabi SS Semnani F Aminzade Z Mehrizi R Gholamnezhad M Armand R. Prevalence, predictors, and clinical relevance of drug–drug interactions in outpatient prescribing: a national cross-sectional study. PLoS One. (2026) 21:e0345076. 10.1371/journal.pone.0345076 41950170 PMC13061183

[30] Gandra S Kotwani A. Need to improve availability of access group antibiotics and reduce the use of watch group antibiotics in India for optimum use of antibiotics to contain antimicrobial resistance. J Pharm Policy Pract. (2019) 12:20. 10.1186/s40545-019-0182-1 31346472 PMC6636108

[31] Antimicrobial Resistance Collaborators. Global burden of bacterial antimicrobial resistance in 2019: a systematic analysis. Lancet. (2022) 399:629–55. 10.1016/S0140-6736(21)02724-0 35065702 PMC8841637

[32] Bansal A Sharma R Prakash R. Adoption of the World Health Organization access, watch reserve index to evaluate and monitor the use of antibiotics at a tertiary care hospital in India. Perspect Clin Res. (2022) 13:90–3. 10.4103/picr.PICR_202_19 35573458 PMC9106137

